# Congenital Chagas disease: Updated recommendations for prevention, diagnosis, treatment, and follow-up of newborns and siblings, girls, women of childbearing age, and pregnant women

**DOI:** 10.1371/journal.pntd.0007694

**Published:** 2019-10-24

**Authors:** Yves Carlier, Jaime Altcheh, Andrea Angheben, Hector Freilij, Alejandro O. Luquetti, Alejandro G. Schijman, Manuel Segovia, Noemie Wagner, Pedro Albajar Vinas

**Affiliations:** 1 Laboratoire de Parasitologie, Faculté de Médecine, Université Libre de Bruxelles (ULB), Brussels, Belgium; 2 Department of Tropical Medicine, School of Public Health and Tropical Medicine, Tulane University, New Orleans, Louisiana, United States of America; 3 Servicio de Parasitología, Hospital de Niños Ricardo Gutiérrez (Centro colaborador en Chagas pediátrico OPS/OMS), Instituto Multidisciplinario de Investigación en Patologías Pediátricas (IMIPP), CONICET-GCBA, Buenos Aires, Argentina; 4 Centro per le Malattie Tropicali, IRCCS Ospedale “Sacro Cuore—Don Calabria,” Negrar (Verona), Italy; 5 Laboratório de Chagas, Hospital das Clínicas, Universidade Federal de Goiás, Goiânia, Goiás, Brazil; 6 Laboratorio de Biología Molecular de la Enfermedad de Chagas, INGEBI-CONICET, Buenos Aires, Argentina; 7 Unidad Regional de Medicina Tropical, Hospital Clínico Universitario Virgen de la Arrixaca, El Palmar (Murcia), Spain; 8 Hôpitaux Universitaires de Genève, Geneva, Switzerland; 9 Department of Control of Neglected Tropical Diseases, World Health Organization, Geneva, Switzerland; Instituto de Ciências Biológicas, Universidade Federal de Minas Gerais, BRAZIL

## Introduction

In 2005, the World Health Organization (WHO) recognized Chagas disease (CD; *Trypanosoma cruzi* infection) as a neglected tropical disease (NTD) [1] and included it into the global plan to combat NTDs [2]. The Target 3.3 of the United Nations Sustainable Development Goals (UN/SDG) aims at ending the epidemics of NTDs by 2030 [3]. Mother-to-child (congenital/connatal) transmission is currently the main mode of transmission of *T*. *cruzi* over blood transfusions and organ transplantations in vector-free areas within and outside Latin America (LA). Based on recent demonstrations that congenital transmission can be prevented [4–7], WHO has shifted its objective, in 2018, from control to elimination of congenital CD (cCD) (road map reference documents in preparation).

This article summarizes the recommendations of the WHO Technical Group on “Prevention and Control of Congenital Transmission and Case Management of Congenital Infections with *Trypanosoma cruzi*” (WHO, Department of Control of Neglected Tropical Diseases). It updates and completes the recommendations previously published in 2011 by the Technical Group [8]. These consensual recommendations derive from discussions at technical meetings convened by WHO in Murcia (Spain) on 9–10 October 2018 (II WHO Technical Consultation on Control of Congenital Chagas disease in nonendemic countries, and specific meetings of the Technical Group).

## Preliminary considerations on congenital transmission of *T*. *cruzi* infection

Congenital transmission of *T*. *cruzi* infection is considered as such when (i) a neonate is born to an infected mother (i.e., with positive serology and/or *T*. *cruzi* parasites circulating in the blood) and (ii) *T*. *cruzi* parasites are identified in neonate blood at or after birth or (iii) specific antibodies not of maternal origin are detected after birth and (iv) previous transmission to infant by vectors and/or blood transfusion has been ruled out [8].

Mother-to-child transmission of CD can occur in infected women living in LA or outside of LA if they have previously lived in LA endemic area and/or were born in LA or whose mothers were born in LA. It can be recurrent at each pregnancy and from one generation to another, leading to family clustering of congenital transmission cases [9]. This pattern of transmission facilitates uncontrolled spread of CD over time, especially in urban areas in both endemic and not endemic countries.

cCD remains an important global and neglected public health problem. Information on prevalence in pregnant women and incidence of congenital cases is frequently lacking, and epidemiological data mainly derive from estimations. WHO estimates that 1,125,000 women in fertile age are infected with *T*. *cruzi* in LA, with an incidence of congenital infection of 8,668 cases/year [10]. The mean maternal–fetal transmission rate in chronic CD (the most frequent phase of infection) in LA is estimated to 4.7% [11]. The number of babies that would need to be tested per year has been estimated from 158,000 to 214,000 (mostly in Argentina, Brazil, Bolivia, and Mexico) [12].

Through migrations from LA, an estimated 40,000 infected women of childbearing age reside in the United States, where 60 to 315 congenital infections are expected to occur annually [13]. In Europe, the annual number of infected pregnant women has been estimated between 1,347 and 2,521, with 20 to 184 cases of congenital transmission [14]. Cases of cCD have been reported mainly in Spain (approximately 90%) but also in Sweden, Switzerland, and more recently in Italy, the US, Canada, and Japan [15,16].

Historically, cCD was associated with high levels of neonatal morbidity and mortality, although nowadays, it is clear that most cases are asymptomatic [13, 15]. Some cCD cases can present nonspecific symptoms, if any, as seen in other intrauterine or perinatal infections (like *Toxoplasma gondii*, *Treponema pallidum*, rubella virus, cytomegalovirus, HIV, herpes simplex virus, and parvovirus infections). Therefore, detection of *T*. *cruzi* congenital infection should rely on easy-to-use and point-of-care diagnostic tools [17].

Congenital *T*. *cruzi* infection is an acute infection in newborns that should be treated with antiparasitic therapy. Left untreated, the infection can progress to chronic CD later in life, with a drop in the cure rate.

### Targeted populations for prevention and control of congenital transmission of *T*. *cruzi*

In order to eliminate congenital transmission of *T*. *cruzi*, efforts should be focused on 5 population groups living within or outside of LA (as defined previously), namely the following:

1. Girls and female adolescents (pre–conceptional phase).
2. Women of fertile age not yet pregnant (childbearing age), by detecting *T*. *cruzi* infection and treating those who are infected (aiming to prevent congenital transmission and reduce the pool of infected population) [4–7]. Control of vectors in endemic areas of LA and blood transmission in endemic as well as nonendemic areas have to be pursued to reduce the risk of infection and the reservoir of infected women.
3. Pregnant women, by antenatal screening for infection performed before or even when entering in maternity. Among those who are infected, there is no way to identify in advance those who will transmit the infection to their offspring, and there are no means of preventing such congenital infection at that moment (antiparasitic treatment is not recommended during pregnancy, see “Treatment of infected girls or adults”). Infected mothers should be treated after delivery and lactation period (see “Treatment of infected girls or adults”) (aiming to prevent recurrent congenital transmission in successive gestations and reduce the pool of infected population).
4. Neonates/infants born to infected mothers by investigating congenital infection and treating and following up all detected positive cases (aiming to control congenital infection, reduce the pool of infected population, and to prevent further congenital transmission in girls) [8].
5. Relatives and other children born to infected mothers (siblings) by investigating their infection status and treating all positive cases (aiming to reduce the pool of infected population from index cases).

## Recommendations for laboratory diagnosis

### Detection of chronic infection in women

As previously recommended by WHO [18], it can be performed using at least 2 of the conventional serological tests (indirect hemagglutination assay; indirect immunofluorescence assay; or ELISA, based on crude or recombinant antigens) in order to increase diagnosis accuracy. These tests are generally available at low cost in primary healthcare facilities. In cases of discrepancies among 2 tests, serology will be repeated in a new sample, and if results remain inconclusive, a third test (e.g., western blot) should be done. The highly sensitive chemiluminescent immunoassay (CMIA) [19] can be used for screening as a stand-alone test (although it remains logistically and economically prohibitive for resource-limited settings), but positive results should be confirmed by conventional serological tests. All these diagnostic tests are not point-of-care tests (they must be performed in a laboratory), and results are not immediately available.

Rapid diagnostic tests detecting antibodies using distinct antigen sets are easy to use (whole blood based, need neither electricity nor cold chain, and results are available within an hour [20]). Used combined to limit discordant results [21], these screening tests could be of help for pregnant women entering maternity facilities for delivery without previous serological diagnosis or at primary healthcare facilities in rural or remote areas. However, by waiting for field validation at large scale, they can be considered as “uptaking” tests in order to prevent losing the patient and also need confirmation with standard tests.

Molecular tests (see “Detection of congenital infection in neonates”) have low sensitivity in the chronic phase of infection and can only be used as complementary diagnosis methods to serological screening.

### Detection of congenital infection in neonates

It can be performed by detecting living parasites in the umbilical cord or, preferably, venous blood of the newborn. Parasitological techniques concentrate parasites by centrifugation using capillary tubes (microhematocrit test) or Eppendorf tubes (“microstrout” method). Parasites are investigated by microscopic examination of the blood buffy coat [8]. Direct detection of the parasite in blood is facilitated by the fact that *T*. *cruzi* has a characteristic movement pattern and a relatively large size that allow its visualization by routine microscopy. If the test is negative at birth, it should be repeated at one month of age, when the peak of parasitemia is usually observed [7, 22–24]. These parasitological tests offer undisputable and definitive diagnosis of infection and are considered as gold standards. However, they require time, prompt processing of the sample (within 24 hours), adequately trained laboratory personnel, and quality controls—factors that can influence accuracy and reliability of the results [13, 23]. Other parasitological tests, based on multiplication of parasites, as hemoculture, are not routinely used for diagnosis of congenital cases. They are “in house” made, require huge equipment with biological protection, in addition to well-trained personal, and demand several weeks to get a result and need several milliliters of blood only available when using cord blood [24, 25]. Therefore, more sensitive and automated tests are needed for early detection of very low levels of *T*. *cruzi*, particularly when transmission occurs in the last period of pregnancy, close to or even at birth.

Molecular methods are another approach for early detection of infection in the blood. They are promising and increasingly used (particularly in Europe), though they present some limitations. They are still economically prohibitive for routine screening in resource-limited settings [26]. False positive results may occur, particularly if samples are collected from umbilical cord blood at birth [15, 27]. They lack standardization, leading to various sensitivity levels according to the centers, and quality control programs are still not implemented enough [28]. As a consequence, molecular methods require stronger and wider clinical validations before being considered as gold standards to diagnose congenital infection. Thereby, the following factors should be taken into account: the best timing of blood sampling (1 to 3 month[s] after birth rather than at birth, unless in presence of a clinically ill newborn), the number of samples to be taken, the sample collection process (EDTA, guanidine EDTA, filter paper, blood clot), the DNA extraction procedure, the DNA target (satellite DNA and/or kinetoplast DNA), the type of method (standard PCR or real-time PCR, requiring slightly more complex equipment and higher cost), the quality control, and the biological standards [28–34]. Therefore, currently, molecular tests can be considered as uptaking tests in order to prevent losing the patient during the follow-up, when parasitological techniques are not available/reliable for logistical/organizational constraints or lack of skilled personnel. The loop-mediated isothermal amplification (LAMP) appears as a promising novel diagnostic test because it uses a single tube, it does not require a thermocycler, and results are viewable by the naked eye within an hour. Nevertheless, it requires further validation for the diagnosis of congenital infection [35–37].

Histological examination of placenta or molecular detection of *T*. *cruzi* DNA in the placental tissues have limited sensitivity, and placental involvement does not closely correlate to fetal infection [38, 39].

### Detection of congenital infection in infants by serological tests when maternal antibodies have disappeared

Serological tests for infants should be used as for mothers (see “Detection of chronic infection in women”). Detection of *T*. *cruzi*–specific antibodies in infants older than 10 months of age (i.e., after elimination of passively transferred maternal antibodies) indicates a congenital infection (when previous transmission by vectors and blood transfusion has been ruled out). By contrast, a negative serological result in infants (born to infected mother) at 10 months of age or thereafter indicates an absence of congenital infection. Such detection of *T*. *cruzi*–specific antibodies in infants is a gold standard for diagnosis of cCD [8]. It should be performed in all infants born to infected mothers if previous tests (parasitological and/or molecular methods) were negative or if no screening tests were done before. However, testing infants only at 10 months old delays diagnosis and treatment and increases the risk of loss to follow-up.

Other tests detecting antibodies in blood, such as trypomastigote excreted-secreted antigens blots IgM or shed acute-phase antigens (SAPA) ELISA IgG (detecting SAPA within the first 3 months of infection [22, 40]), are not commercially available and need to be validated at a larger scale.

Health systems should evaluate and implement the strategies that facilitate the earliest possible diagnosis of congenital infection, taking into account the frequent poor compliance of the mothers to attend follow-up visits to the health centers.

## Recommendations for treatment

### Treatment of infected neonates and infants

Cases of congenital *T*. *cruzi* infection should be treated as soon as the diagnosis has been confirmed. The current experience of expert clinical groups in treating congenital *T*. *cruzi* infection confirms that (i) both benznidazole (BZ) and nifurtimox (NF) can be used to treat congenital cases; (ii) the recommended dose of BZ in infants is 5 to 7 mg/kg per day, divided in 2 doses, and that of NF is 10 to 15 mg/kg per day, divided in 3 doses; (iii) such doses can be administered orally in one dose in low-weight neonates; precautions should be taken to obtain appropriate dosage of active drug, particularly with no dispersal tablets (see point v), which have to be crushed and used as a suspension; (iv) the recommended duration of treatment is 60 days and should not be <30 days [41]; (v) BZ is available in dispersal tablets of 12.5 mg and NF in tablets of 120 mg (dispersal tablets of 30 mg should be available proximately). Both drugs can be obtained free of charge near WHO (NF for patients of all ages and BZ for patients aged under 19 years).

Current expert field experience indicates that treatment is highly effective with lower adverse events than those described in adults [42]. Cure rates, as evaluated by conventional serology, are over 90% in infants treated during the first year of age. Randomized comparative clinical trials in infants or trials combining BZ or NF with new chemical entities are strongly desired to adjust/optimize the dose and/or duration of treatment.

Treatment follow-up is recommended by parasitological and/or molecular tests in the weeks after the treatment onset for neonates displaying parasitemia. After treatment completion, patients need to be followed every 6 months with quantitative serological tests. The patient is considered cured when serology becomes negative.

### Treatment of infected girls or adults

They should be treated with BZ or NF according to the standard recommendations of WHO [18]. However, antiparasitic treatment is contraindicated during pregnancy, because the risks of using the available medicines BZ and NF on the fetus are unknown, and the risk of adverse reactions is high in adults [43]. Infected mothers will be therefore treated after delivery and the lactation period to avoid interruption of lactation as a result of such possible adverse reactions.

## Other recommendations

Clinical evaluation and follow-up are required for mothers displaying cardiac and/or digestive forms of CD [18]. It is recommended that infected mothers do not donate blood and that cord blood from neonates born to infected mothers is not used for bone marrow transplantation, due to the risk of inducing an acute CD in an immunosuppressed recipient [44].

CD should be systematically investigated in relatives and other children born to infected mothers (serological diagnosis), and positive cases should be clinically evaluated and treated accordingly.

### Recommendations for healthcare systems and public health organizations

The barriers to healthcare access should be reduced to facilitate the diagnosis of CD and avoid loss to follow-up in the target populations.

Awareness, education, and training of healthcare workers at different levels on prevention and control strategy of cCD have to be improved, as well as the cooperation among the primary healthcare, hospital, and public health actors.

Information, education, and communication on cCD should be strengthened within community and patient associations with commitment of community health workers near the affected population.

Detection of cCD has to be integrated within programs aiming to detect other congenital infections (e.g., HIV, syphilis, hepatitis B, and the other intrauterine or perinatal infections mentioned previously) [45].

Epidemiological information systems and notification and monitoring of cases and health risks (as population profiles rapidly change due to their mobility in times of economic constraints [46]) have to be promoted and implemented in order to obtain more accurate information on prevalence of infection in pregnant women and incidence of congenital cases and verify the process of interruption of transmission. This can be done through the recently updated WHO information system to control/eliminate NTDs (WISCENTD) and its WHO integrated data platform (WIDP) [47].

Control strategy of cCD has been shown to be cost saving [48–51], fully justifying its integration at the public health level.
